# Survey of the patients' perspectives and preferences in adopting telepharmacy versus in-person visits to the pharmacy: a feasibility study during the COVID-19 pandemic

**DOI:** 10.1186/s12911-022-01834-5

**Published:** 2022-04-13

**Authors:** Khadijeh Moulaei, Mostafa Shanbehzadeh, Kambiz Bahaadinbeigy, Hadi Kazemi-Arpanahi

**Affiliations:** 1 Medical Informatics Research Center, Institute for Futures Studies in Health, Kerman University of Medical Sciences, Kerman, Iran; 2 Department of Health Information Technology, School of Paramedical, Ilam University of Medical Sciences, Ilam, Iran; 3 Department of Health Information Technology, Abadan University of Medical Sciences, Abadan, Iran; 4 Student Research Committee, Abadan University of Medical Sciences, Abadan, Iran

**Keywords:** COVID-19, Coronavirus, Telemedicine, Telepharmacy, In-person visits

## Abstract

**Background:**

Following the coronavirus disease 2019 (COVID-19) pandemic, the health authorities recommended the implementation of strict social distancing and complete lockdown regulations to reduce disease spread. The pharmacists quickly adopted telemedicine (telepharmacy) as a solution against this crisis, but awareness about this technology is lacking. Therefore, the purpose of this research was to explore the patients' perspectives and preferences regarding telepharmacy instead of traditional in-person visits.

**Methods:**

An electronic questionnaire was designed and sent to 313 patients who were eligible for the study (from March to April 2021). The questionnaire used five-point Likert scales to inquire about motivations for adopting telepharmacy and in-person visits, their perceived advantages and disadvantages, and the declining factors of telepharmacy. Finally, the results were descriptively analyzed using SPSS 22.

**Results:**

Of all 313 respondents, a total of 241 (77%) preferred appointments via telepharmacy while 72 (23%) preferred in-person services. There was a significant difference between the selection percentage of telepharmacy and in-person services (chi-square 91.42; *p* < 0.0001). Preference bout the telepharmacy system versus in-person visits to the pharmacy was associated with factors such as "reducing the incidence of contagious disease" (4.41; ± 0.78), "spending less time receiving pharmaceutical services” (4.24; ± 0.86)), and “traveling a shorter distance for receiving pharmaceutical services" (4.25; ± 0.86). "Reducing costs" (90.87%), "saving time" (89.21%), and "reducing the incidence of contagious disease" (87.13%) were the most important reasons for choosing telepharmacy services. Also, “face-to-face communication with the pharmacist” (25%), “low internet bandwidth” (25%), and “reduction of patients' anxiety and the increase of their peace of mind” (23.61%) were the most important reasons for choosing in-person visits.

**Conclusion:**

Survey data indicate that most participants are likely to prefer the use of telepharmacy, especially during crises such as the current COVID-19 pandemic. Telepharmacy can be applied as an important means and a crucial service to lessen the load on healthcare organizations and expand drug supply shelters in pharmacies. However, there are still substantial hurdles to overcome in order to successfully implement the telemedicine platform as part of mainstream practice.

**Supplementary Information:**

The online version contains supplementary material available at 10.1186/s12911-022-01834-5.

## Background

The coronavirus disease (COVID-19) presents mainly mild or moderate manifestations, but sometimes it leads to severe and even fatal outcomes. This pandemic has so far remained widespread and has become a serious international health concern [1, 2]. The coronavirus proved to be very contagious and fatal compared with its antecedents, including severe acute respiratory syndrome coronavirus 1 (SARS-CoV) and Middle East respiratory syndrome-related coronavirus (MERS-CoV) [3, 4]. The COVID-19 outbreak has overwhelmed the public health and healthcare systems worldwide, pushing them to the brink of prominent collapse via the escalating requests for life-saving medications, the troubling blockades in the patient care access, and the aggravation of the scarcity of healthcare resources and providers [5, 6]. Today, in the midst of a pandemic that has overburdened physicians and nurse practitioners, pharmacists can make crucial contributions to public health, particularly in disease inhibition, control, and containment [7, 8].

Pharmacists are a strategic line of public health response during this pandemic and have performed a fundamental role as frontline healthcare workers in disease mitigation and control [9]. During the current pandemic, their responsibilities extend beyond the dispensing and supplying of medicines. They are required to offer pharmaceutical care for COVID-19 patients, especially for high-risk populations such as the elderly and those who have underlining conditions [7, 10]. They accompany the patients to provide either direct or indirect interventions and contribute to the medication supply chain management, help to improve patients’ drug adherence, counsel the patients, and educate and inform the public about the most recent and effective supportive drugs and vaccines available for treatment or immunization [8, 11, 12].

During the COVID-19 pandemic, when strict physical distancing and movement restrictions were mandated and an unprecedented surge in healthcare demands was observed, telemedicine became the preferred care delivering mode, enabling electronic counseling and diminishing the risk of disease transmission by reducing face-to-face appointments. In response to COVID-19, more individuals use online pharmacies and more countries encourage people to buy medicines online [13]. In this regard, some nations have officially allowed the extension of the role of virtual consultations by pharmacists and remote dispensing using telecommunication technologies [14–16].

Many healthcare authorities and policymakers advocated for the use of telemedicine to address some challenges that healthcare systems are facing in the battle against the COVID-19 crisis [17–19]. Telemedicine refers to a mode of healthcare delivery to patients through information and communications technology (ICT)-based visits and remote monitoring. It consists of a variety of electronic communication methods and digital tools to allow healthcare workers to interact with patients “remotely” [20–22]. Video and audio media, sometimes accompanied by web- or app-based interfaces, both synchronous and asynchronous, are common modalities through which telemedicine can be provided [23, 24]. Similar to most other telemedicine subclasses, telepharmacy is getting more attention during the COVID-19 pandemic. As defined by the world health organization (WHO) and the US Medical Institute, telepharmacy is the distant delivery of all aspects of pharmaceutical care through ICTs. “It covers a wide range of pharmaceutical services, such as drug prescribing, dispensing, counseling, adherence, monitoring, education, order analysis, and provision of clinical services” [25, 26].

Given the government-imposed severe restrictions on social interactions and travel during the COVID-19 pandemic, telepharmacy offers a great potential for eliminating face-to-face visits. It diminishes the pressure on healthcare systems by providing teleservices for COVID-19 carriers [27–29]. To warrant the success of any telemedicine platform, the patients’ satisfaction and their preferences should be recognized [30]. Hence, the purpose of this study was to examine patients’ attitudes and preferences by evaluating their willingness to adopt telepharmacy services during the COVID-19 pandemic rather than in-person visits to the pharmacy. Moreover, the authors specifically survey how patients perceive telepharmacy and what facilitators and barriers affect their ability to engage in a telepharmacy program.

## Methods

### Study design

This cross-sectional study was performed in 2021. We conducted a survey to gain a reflective understanding of the participants’ preferences and views regarding the use of telepharmacy versus in-person visits to the pharmacy during the COVID-19 pandemic. For this purpose, first, a literature review was conducted in scientific databases to become familiar with and understand the knowledge and concepts in the field of telemedicine and particularly telepharmacy. Then, based on the results of the literature review, an electronic questionnaire was designed to collect data from eligible participants.

### Study population and sample

During the study period, a total of 900 patients with different diseases were referred to six specialized clinics at Golestan Hospital affiliated with Jondishapour University of Medical Sciences (Ahvaz, Iran). An available sampling method was applied to select the study participants and all 900 patients were invited to the study. Using the patients' medical records, their contact information was extracted, and an electronic invitation was sent to 900 patients to participate in the study. The invitation was designed using the fotojet website (https://www.fotojet.com/features/photo-card/invitation.html) and sent to the participants via social networks, such as WhatsApp and Telegram, as well as Email. The invitation explained the purpose of the study, emphasized the voluntary and anonymous nature of the survey, and outlined the extent of confidentiality. The questionnaires were distributed through social network apps (such as WhatsApp and Telegram) and Email. Overall, 418 patients agreed to participate in the study. Finally, according to the inclusion and exclusion criteria, 313 participants remained.

### Inclusion and exclusion criteria

**Fig. 1:**
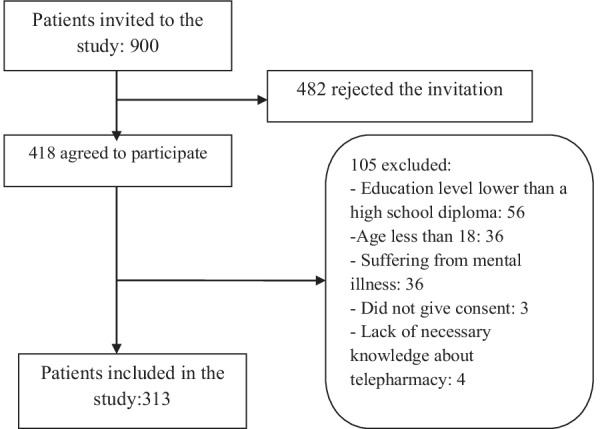
Diagram of participant selection

The present study included patients who did not suffer from mental and cognitive disorders, had at least a high school education, and were at least 20 years old. In addition, the participants had to be familiar with the concept of telepharmacy and its advantages. They also had to be sick and should have given consent to participate in the study (see Fig. 1). It should be noted that after 418 patients agreed to be included in our study, we sent the inclusion criteria and consent form along with a questionnaire to the patients. We asked patients to leave the study if they did not meet the inclusion and exclusion criteria or did not approve the consent form. We also asked them to specify for us what inclusion criteria they do not have.

### Questionnaire development

Data collection was performed using a researcher-made questionnaire. To develop the questionnaire, at first, three databases including PubMed, Scopus, and Web of Science were searched, and 60 articles were retrieved from these databases. The titles and abstracts of the articles were reviewed by researchers (HKA and KHM). All valid articles were reviewed and consequently approved by KB and MSH. Then, the full text of the articles was reviewed by KHM and HKA to extract the required information. Finally, based on inclusion and exclusion criteria, 28 articles [7, 11, 14–16, 27–29, 31–49] were included in the present study and were considered for questionnaire design. Inclusion criteria included articles conducted on humans published in English and articles focusing on telepharmacy. Exclusion criteria included articles not related to telepharmacy. Moreover, books, letters to the editor, and conference abstracts were not included in the study.

The full text of all 28 articles was studied by two researchers, and then all the necessary data elements for designing the questionnaire were extracted from the articles. A data extraction form (including article reference fields, the study objective, and data elements) was used to extract the data elements. Afterward, based on the extracted data elements, the final questionnaire was developed and administered in the Persian (Farsi) language, which is the national language in Iran.

Based on the data elements extracted from the literature review, several ideas were identified to formulate the survey questions used in the current study. After identifying the initial framework of the questionnaire, the questions were reviewed by four experts in the field of pharmacy and medical informatics to provide the necessary changes and establish face and content validity analysis. Afterward, the reliability of the questionnaire was evaluated during the pilot testing stage by collecting data from 40 participants (with a 10-day interval) who were not included in the study sample. A Cronbach's alpha was calculated for internal consistency and scale reliability among related questions. Cronbach's alpha values of the resilience domains ranged from 0.71 to 0.835.

After confirming the validity and reliability, the questionnaire was designed electronically using the Google Forms tool (Additional file 1).

The questionnaire consisted of three parts: Part 1 collected information on participants' demographic characteristics such as age, sex, marital status, literacy, and similar factors. Part 2 contained details related to patients’ views towards telepharmacy versus in-person visits to the pharmacy (23 questions). Part 3 had one question on whether the participants preferred telepharmacy or in-person visits to the pharmacy.

The responses for each question were recorded on a five-point Likert scale ranging from strongly agree to strongly disagree. The score for each response ranged from 5 (strongly agree) to 1 (strongly disagree). In addition, a telephone number was included in the questionnaire so that the participants could contact the research team with any queries or uncertainties. To fill the electronic questionnaire, all the questions were defined as necessary; therefore, all of the participants answered all the questions.

### Data collection

Before sending the questionnaire link to the participants, 10 questionnaires were filled out by the participants to estimate the time required to complete the questionnaire. It was stated in the questionnaire that an average of 10–12 min is needed to respond to the questions. From March 10 to April 10, 2021, the electronic link to the questionnaire was sent to the participants. Along with the forward link, the participants received an educational guideline regarding the content of the questionnaire, research goals, and how to complete the questionnaire. Before filling out the questionnaire, we asked the participants to sign the online consent form attached to the first page of the online survey to confirm their agreement to participate in the study.

After the participants answered the questions, the responses were stored on the  Google Forms. Finally, all the participants' responses were retrieved in an Excel file and imported to SPSS 25.

### Data analysis

For each question, a variable was defined by some features including name, data type, data length, label, and scale. The demographic characteristics of the participants were examined based on frequency and percentage. In addition, descriptive statistics (percentage, mean, and standard deviation), medians, interquartile ranges (IQRs), and the chi-square test were used to analyze other parts of the questionnaire. All statistical analyses were performed using SPSS (IBM SPSS statistics version 25; IBM Corp., Armonk, NY, USA, 2015).

### Ethical considerations

This study was approved by the ethics committee board of Abadan University of Medical Sciences (Ethics code: IR.ABADANUMS.REC.1400.019). Before participation in the study, electronic informed consent was obtained from the participants. The patients were also informed that they would remain anonymous and that the information they provided would be handled confidentially. All respondents gave their informed consent and the data were kept on a secure device.

## Results

Table 1 shows the demographic information of the patients participating in the study. According to the following table, most of the participants were women (53%), had a bachelor's degree (44.7%), and resided in the city (87.5%). Moreover, most participants had underlying cardiovascular diseases.

**Table 1:** Demographic and clinical characteristics of the study participants (n = 313)

Variables	Frequency	Percentage
*Sex*		
Male	147	47
Female	166	53
*Age*		
25–35	120	38.28
36–45	100	31.9
46–55	80	25.52
> 55	13	4.147
*Education level*		
Diploma	85	27.2
Associate degree	33	10.5
Bachelor	140	44.7
Master	30	9.6
Ph.D. and above	25	8
*Residence type*		
City	274	87.5
Village	39	12.5
*Disease type*		
Cardiovascular diseases	105	33.49
Respiratory diseases	80	25.52
Eye diseases	41	13.07
Otorhinolaryngology diseases	30	9.57
Psych neurological diseases	22	7.01
Gynaecological diseases	18	5.74
Genetic disorders	12	3.82
Rare diseases	5	1.59
*Duration of the disease (years)*		
1–10	180	57.42
11–20	100	31.90
≥ 20	33	10.52

Table 2 presents the perspectives and beliefs of the participants about the telepharmacy system versus in-person visits to the pharmacy. Based on this table, among the different beliefs (23 sub-themes), "reducing the incidence of contagious disease" (4.41 (± 0.78)), "spending less time receiving pharmaceutical services” (4.24 (± 0.86)), and “traveling a shorter distance for receiving pharmaceutical services” (4.25 (± 0.86)) had the highest means. On the other hand, “insufficient technological literacy and skills” (3.65 (± 1.02)), “easy communication and interaction with pharmacists” (3.64 (± 1.09)) and “increased adherence to medication” (3.50 (± 1.01)) in telepharmacy compared with face-to-face counseling obtained the lowest scores. However, despite the low means of these three beliefs compared to the other views, these scores are still very high (above 3).

**Table 2:** Patient’s beliefs regarding the implementation of telepharmacy versus in-person visits

Themes	Patient’s views and beliefs (sub-themes)	Mean (± SD)	Interquartile range (IQR)		
			Q1	Median	Q3
Pharmaceutical services	Reducing the incidence of contagious disease by receiving remote pharmaceutical services	4.41 (± 0.78)	4.00	5.00	5.00
	Improve the information security and confidentiality	4.05 (± 0.91)	4.00	4.00	5.00
	Legal tracking of medication errors	3.87 (± 0.97)	3.00	4.00	5.00
	More effective reporting of drug side effects to pharmacists and physicians	3.83 (± 1.01)	3.00	4.00	5.00
	Increase cooperation and interactions between doctor and pharmacist	3.82 (± 0.97)	3.00	4.00	5.00
	Delivering better and easier pharmaceutical services (distribution and prescription)	3.80 (± 1.01)	3.00	4.00	5.00
	Providing better medication recommendations	3.79 (± 1.01)	3.00	4.00	5.00
	Receive drug services all the time and around the clock	3.79 (± 0.51)	4.00	4.00	4.00
	Reducing medication errors, allergy and drug interactions	3.71 (± 1.04)	3.00	4.00	4.00
	Increase adherence to medication	3.50 (± 1.01)	3.00	3.00	4.00
Ease and low cost of use	Travel shorter distance for receiving pharmaceutical services	4.25 (± 0.86)	4.00	4.00	5.00
	Spend less time receiving pharmaceutical services	4.24 (± 0.86)	4.00	4.00	5.00
	Compensate the lack of physicians or pharmacists and facilities in rural areas	4.04 (± 0.92)	4.00	4.00	5.00
	Easy, accurate, and real-time documentation	3.97 (± 0.83)	3.00	4.00	5.00
	Easier and faster access to clinical and pharmaceutical information by pharmacists and physicians	3.96 (± 0.85)	3.00	4.00	5.00
	Reduce costs	3.86 (± 0.97)	3.00	4.00	5.00
	Reduce patients' anxiety and stress	3.83 (± 1.02)	3.00	4.00	5.00
	Easy exchange of information with pharmacists and physicians	3.82 (± 1.10)	3.00	4.00	5.00
	Easier payment	3.79 (± 0.96)	3.00	4.00	5.00
	Better and easy scheduling of revisits	3.77 (± 0.93)	3.00	4.00	4.00
	Easy communication and interaction with pharmacists	3.64 (± 1.09)	3.00	4.00	4.00
Technology access and use	Limited access to the internet connection / Low bandwidth	4.00 (± 0.89)	3.50	4.00	5.00
	Insufficient technological literacy and skills	3.65 (± 1.02)	3.00	4.00	4.00

For the three themes of "pharmaceutical services", "ease and low cost of use", and "technology access and use", the highest mean was related to "reducing the incidence of contagious disease" followed by "traveling a shorter distance for receiving pharmaceutical services" and "limited access to internet connection/low bandwidth".

Of the 313 participants in the study, 241 (77%) preferred to use telepharmacy, while 72 (23%) desired to receive pharmaceutical in-person services. In addition, the results of the chi-square test revealed a significant difference between the selection percentage of telepharmacy and in-person services. The percentage of participants who chose telepharmacy was higher (chi-square 91.42; *p* < 0.0001).

Table 3 presents the different reasons why the participants who preferred telepharmacy chose these services. According to this table, out of the 20 reasons for choosing telepharmacy services, "reducing costs" (90.87%), "saving time" (89.21%), and "reducing the incidence of contagious disease" (87.13%) were the most important ones.

**Table 3:** Patients’ reasons for choosing telepharmacy services

Row	Patient reasons for choosing telepharmacy services	Frequency	Percent
1	Reducing costs (cost-effectiveness)	219	90.87
2	Saving time	215	89.21
3	Reducing the incidence of contagious disease	210	87.13
4	Reduce patients' stress or anxiety and increase their peace of mind	205	85.06
5	Receive pharmaceutical services and advice anytime, anywhere	189	78.42
6	Easier exchange of information with pharmacists and physicians	180	74.68
7	Easy access to pharmaceutical services especially for deprived and rural areas	169	70.12
8	Diminish congestion at the pharmacies	164	68.04
9	More confidentiality, security and privacy	160	66.39
10	Reduce Medication Errors (In dose adjustment, administration and distribution of drugs)	159	65.97
11	Easier legal follow-up	140	58.09
12	Access to patients' treatment history	138	57.26
13	Reduce chaos in the pharmacies	82	34.02
14	Shorten waiting time at the pharmacy	55	22.82
15	Get more comprehensive and accurate information about patients	43	17.84
16	Registration of drug history in the system	41	17.01
17	Increase cooperation between physicians, pharmacists and patients	8	3.31
18	Less exposure to air pollution	5	2.07
19	Provide educational services related to how to take medicines	3	1.24
20	More accurate and customized prescription	2	0.82
Total number of responses based on the opinion of 241 patients		2387	

Because each of the 241 participants could write more than one answer, the total number of replies was 2387. Also, the percentages are calculated out of the total number of 241 participants

Table 4 also shows 11 different reasons for choosing in-person visits to the pharmacy. Among these reasons, “face-to-face communication with the pharmacist” (25%), “low internet bandwidth” (25%), and “reducing patients' stress or anxiety and increasing their peace of mind” (23.61%) were the most important ones.

**Table 4:** Patients’ reasons for choosing in-person visits to the pharmacy

Row	Patient reasons for choosing in-person services	Frequency		Percent
1	Face-to-face interaction with the pharmacist	18		25
2	Low internet bandwidth	18		25
3	Reducing patients' stress or anxiety and increasing their peace of mind	17		23.61
4	Reluctance to use new advanced technologies	15		20.83
5	Easier access to pharmaceutical services	14		19.44
6	Get better medical advices	13		18.05
7	Get quick and convenient answers	11		15.27
8	Lack of sufficient knowledge about the concept of telepharmacy and its capabilities	9		12.5
9	More privacy and confidentiality	8		11.11
10	Easy communication and interaction with pharmacy staff	5		6.94
11	Easier receive of prescribed drugs	5		6.94
Total number of responses based on the opinion of 72 patients		133		

Because each of the 72 participants could write more than one answer, the total number of answers was 133. Also, the percentages are calculated out of the total number of 72 participants

## Discussion

In this study, we examined patients' perspectives and preferences in adopting telepharmacy versus in-person visits to the pharmacy. The present study found that 77% of the participants preferred telepharmacy while only 23% of them favored in-person visits. "Face-to-face communication with the pharmacist", "low internet bandwidth", and "reducing patients' stress or anxiety" were the most important reasons why the participants preferred in-person visits to the pharmacy. Moreover, the results showed that using telepharmacy has allowed us to continue care delivery while keeping patients and pharmacists safe during the pandemic. In addition, it has become an efficient solution for providing a convenient and economical way to access pharmaceutical care services remotely. The most important reasons for using telepharmacy in the present work were the protection of patients and pharmacy staff, reduction of the COVID-19 burden in the general population, cost-effectiveness, and improving adherence to drug treatments. Similarly, prior evidence demonstrates that telemedicine has been a practical, cost-effective, and safe means to offer pharmacy services and to improve patient care quality [14, 35, 45]. Numerous pharmacy services can be delivered over telepharmacy; nonetheless, the question arises whether the quality of virtual care is on par with that of in-person visits to the pharmacy from the patients’ perspectives [7, 11, 36, 46].

Previous research on telepharmacy has investigated the various factors that affect the implementation and use of telephramacy services from the perspective of the health care system during the COVID-19 pandemic. In most of the reviewed studies, the study populations, including pharmacists [14, 33, 34, 44, 47], customers, patient [35] and physicians [50] welcomed the implementation of the telepharmacy for teleprescription [14, 28, 46–48], teleconsultation [14, 28, 31–34], and remote dispensing purposes [28, 31, 33, 44, 46–48]. The importance of telephramacy has multiplied during the COVID-19 pandemic when social distancing restrictions challenge the continuity of medication management (CMM) programs. Therefore, in all studies that have investigated telepharmacy in the COVID-19 era from the users’ point of view, its necessity has been emphasized. For example, Muflih et al. [44] analyzed pharmacists' attitudes towards the clinical benefits of telepharmacy and identified the challenges regarding its use during the COVID-19 pandemic. The majority of pharmacists had positive attitudes towards the advantages of telepharmacy including timely feedback (91%), customized and dynamic drug therapy (82.2%), and frequent and accurate communication with the healthcare team (77%). On the other hand, financial problems (76.4%) and the lack of sufficient evidence-based studies (70.8%) were identified as the top challenges [44]. Ibrahim et al. also stated the top factors associated with telepharmacy success in the COVID-19 pandemic from the pharmacists’ perspective. In their study, the two important advantages of telepharmacy over in-person visits were reported to be decreased errors in terms of prescription (up to 3.62%), dispensing (up to 6.35%), and counseling (up to 1.07%), and increased patient access to pharmaceutical care (up to 73%) [42]. These findings were consistent with those of the study by Casey et al. [49] in which a lower rate of medication dispensing errors (MDEs) was reported following the implementation of telepharmacy in rural hospitals. Goitia et al. [34] surveyed the implementation aspects of telepharmacy in a total of 185 hospitals in Spain during the COVID-19 pandemic from the patients’ and pharmacists’ perspectives. About 87.6% of the hospitals implemented tele-consultation services before dispensing and 59.6% offered full delivery telepharmacy services, including tele-prescription, tele-counseling, and remote dispensing. The results showed that an extensive implementation of telepharmacy services led to continuity of care (82.3%), drug consistency (79.2%), and the increase of the quality of medications (78.8%) for a large number of patients. Ameri et al. [36] and Kilova [40] evaluated pharmacists’ opinions on telepharmacy to determine the obstacles and advantages of establishing telepharmacy systems. In addition, in the studies by Mazrouei [46], Hedima [32], Koster [48], Abdel-Wahab [31], and Martin [28], it was shown that telepharmacy services have several advantages during COVID-19 social distancing and self-quarantine. The most important achievements of telepharmacy according to the studies were improving patients’ interactions with pharmacists [14, 28, 31, 46], enhancing the quality of pharmacotherapy and patient safety [31, 32, 47, 48], the continuation of pharmaceutical therapy [28, 32, 46, 47], and increasing drug compliance and consistency [31, 32, 46]. However, the widespread use of this platform is faced with several challenges, including education and information barriers [14, 31, 32, 46], cultural challenges [31], technical infrastructure, and resource requirements [28, 31, 32, 46, 47], as well as legal and supportive issues [14, 31, 32, 46–48]. Many studies also highlighted patient privacy and confidentiality issues [14, 28, 33, 34, 44, 46].

In most of the reviewed studies, the attitudes of pharmacists regarding the implementation of telemedicine were evaluated. No research has yet assessed the patients’ standpoint, except for a small-scale investigation on a group of HIV patients [51] and a primary study on the application of telepharmacy during the pandemic, both of which demonstrated a high perceived quality of telepharmacy [52]. The participants in our study preferred telepharmacy for receiving pharmaceutical services. Moreover, in the present study, according to the participants' responses, 20 different reasons were determined as the potential motivating factors for choosing telemedicine technologies in order to receive pharmaceutical services. From those criteria, "reducing costs", "saving time", and “reducing the incidence of contagious disease" are considered the key determinants in the intention to adopt telepharmacy. Moreover, among the 11 reasons for choosing in-person visits, the three most important reasons are “face-to-face communication with the pharmacist”, “low internet bandwidth”, and “reduction of patients' anxiety and the increase of their peace of mind”. While many study contributors acknowledged an understanding of telemedicine policy, they were unfamiliar with the term telemedicine. These findings suggest that healthcare authorities should design and execute policies to improve patients’ awareness, perception, and usage of telepharmacy services.

Shifting the method of service delivery is inherently time-consuming. The COVID-19 pandemic accelerates the change process, effectively discarding the usual resistance reasons such as system readiness, adopter features, and implementation barriers. The present work proposes that now is the time to lock in changes such as telepharmacy adoption, which have improved patients’ access to pharmacological services, while also addressing hindrances such as high cost and unclear communication with patients. The patients’ standpoint is essential in the co-design and assessment of such technologies. Research from the viewpoints of physicians and pharmacists would complement these findings and assist in revealing the reasons for the impediments experienced by patients [53].

### Limitations

The research included a small number of participants (n = 313), which limits the generalizability of the results to the entire community population. It is suggested that future studies be conducted with more diverse participants and to explore whether there are any other aspects that could influence interest in and the use of telepharmacy. To increase the generalizability and applicability of the existing findings, other telepharmacy stockholders such as healthy individuals and pharmacists should be considered in future works. Considering that no telepharmacy system has been widely implemented so far, it is also suggested that future studies be conducted on the technical and infrastructural aspects of the implementation of this telemedicine modality. Our research was carried out in a specific place and time (eight hospitals in Iran) with distinct technological, cultural, social, and regional issues. Therefore, the results of this study may not be generalizable to other patients elsewhere. To overcome this limitation, more extensive research should be performed in other countries of the world. In addition, this study was conducted electronically, via social networks, which may imply that the participants who have low technological literacy or those without access to social networks, including older adults, were omitted from the research. This may skew the study sample to mostly include individuals who would be more willing to use telepharmacy rather than those who would struggle to use telepharmacy. However, some participants reported responding on behalf of family members who would not otherwise have completed the survey. On the other hand, due to the prevalence of COVID-19, questionnaires were sent to patients online and through social networks. As a result, patients who were not members of social networks were not included in the study. It is suggested that in future studies, people who do not use social networks be included in the study, and questionnaires be distributed among them in person. Because this survey was performed using social networks, the individuals with less than a high school diploma were excluded from the study, which might have led to a selection bias. This bias leads to the exclusion of individuals with lower educational and potentially socioeconomic levels (who are often disadvantaged). However, the global COVID-19 outbreak and the mandated quarantine in Iran made this research methodology feasible.

## Conclusion

Telepharmacy is expected to be a fundamental means of communication between pharmacists' daily practices. During the COVID-19 pandemic, it significantly helped to protect both patients and pharmacists and decrease the spread of infection. It is found that telepharmacy is safe, convenient, and free of the major interpersonal interactions of the patient–pharmacist relationship. Evaluating the advantages and challenges of the adoption of telepharmacy services from the perspective of users contributes to customizing the implementation of this platform and consequently, reducing its failure. Thus, health authorities should be familiar with these advantages and challenges in order to design and implement a successful telepharmacy system. Additional and larger surveys are required to evaluate the quality of telepharmacy visits to demonstrate that virtual appointments are not inferior to in-person ones and to understand which patient sub-groups are most willing to contribute to telepharmacy. While not everyone prefers telepharmacy appointments over in-person visits, the high approval of telepharmacy by patients is promising for the forthcoming development of this technology.

## Supplementary Information

**Additional file 1.** Patients’ perspectives and preferences in adopting the Telepharmacy versus in-person visits to the pharmacy.

## Data Availability

The datasets used and/or analysed during the current study available from the corresponding author on reasonable request.

## Abbreviations

SARS-CoV: Severe acute respiratory syndrome
MERS-CoV: Middle East respiratory syndrome
SD: Standard deviation
CMM: Continuity of Medication Management
MDEs: Medication dispensing errors
