# Supplementary material for: Survey of the patients' perspectives and preferences in adopting telepharmacy versus in-person visits to the pharmacy: a feasibility study during the COVID-19 pandemic
Source: BMC Med Inform Decis Mak. 2022 Apr 13;22:99. doi: 10.1186/s12911-022-01834-5 (PMC9005615; doi:10.1186/s12911-022-01834-5)
Supplement: Supplementary file 1 — Additional file 1. Patients’ perspectives and preferences in adopting the Telepharmacy versus in-person visits to the pharmacy. [file 12911_2022_1834_MOESM1_ESM.docx]

**Appendix A**

**Patients' Perspectives and Preferences in adopting the Telepharmacy versus in-person visits to the pharmacy**

Dear participant, this questionnaire has been developed in order to determine your Perspectives and Preferences about the adopting the Telepharmacy versus in-person visits to the pharmacy. Please answer each question after reading it.

***Section A: Demographic and clinical characteristics***

1. **What is your age in years? .........**
2. **What is your gender?**

- Female
- Male
- Trans-Female
- Trans-Male

1. **What is your education level?**

- Diploma
- Associate degree
- Bachelor
- Master
- PhD and above

1. **What is your residence Type?**

- City
- Village

1. **What is your Disease?**

- Cardiovascular diseases
- Respiratory diseases
- Eye diseases
- Otorhinolaryngology diseases
- Psych neurological diseases
- Gynaecological diseases
- Genetic disorders
- Rare diseases
- Other............

1. **How long have you had the disease (year)?...........................**

***Section B: Patient’s views and beliefs regarding the implementation of telepharmacy versus in-person visits***

1. **Telepharmacy can reduce the incidence of contagious disease by receiving remote pharmaceutical services.**

- Strongly Agree
- Agree
- Undecided
- Disagree
- Strongly Disagree

1. **Telepharmacy can improve information security and confidentiality.**

- Strongly Agree
- Agree
- Undecided
- Disagree
- Strongly Disagree

1. **Telepharmacy can cause legal tracking of medication errors.**

- Strongly Agree
- Agree
- Undecided
- Disagree
- Strongly Disagree

1. **Telepharmacy can lead to more effective reporting of drug side effects to pharmacists and physicians.**

- Strongly Agree
- Agree
- Undecided
- Disagree
- Strongly Disagree

1. **Telepharmacy can increase cooperation and interactions between doctor and pharmacist.**

- Strongly Agree
- Agree
- Undecided
- Disagree
- Strongly Disagree

1. **Telepharmacy can lead to delivering better and easier pharmaceutical services (distribution and prescription).**

- Strongly Agree
- Agree
- Undecided
- Disagree
- Strongly Disagree

1. **Telepharmacy can lead to providing better medication recommendations.**

- Strongly Agree
- Agree
- Undecided
- Disagree
- Strongly Disagree

1. **Telepharmacy can enable receive drug services all the time and around the clock.**

- Strongly Agree
- Agree
- Undecided
- Disagree
- Strongly Disagree

1. **Telepharmacy can cause reducing medication errors, allergies and drug interactions.**

- Strongly Agree
- Agree
- Undecided
- Disagree
- Strongly Disagree

1. **Telepharmacy can cause travel shorter distance for receiving pharmaceutical services.**

- Strongly Agree
- Agree
- Undecided
- Disagree
- Strongly Disagree

1. **Telepharmacy can cause less time for receiving pharmaceutical services.**

- Strongly Agree
- Agree
- Undecided
- Disagree
- Strongly Disagree

1. **Telepharmacy can compensate for the lack of physicians or pharmacists and facilities in rural areas.**

- Strongly Agree
- Agree
- Undecided
- Disagree
- Strongly Disagree

1. **Telepharmacy can provide easy, accurate, and real-time documentation.**

- Strongly Agree
- Agree
- Undecided
- Disagree
- Strongly Disagree

1. **Telepharmacy can provide easier and faster access to clinical and pharmaceutical information by pharmacists and physicians.**

- Strongly Agree
- Agree
- Undecided
- Disagree
- Strongly Disagree

1. **Telepharmacy can reduce costs.**

- Strongly Agree
- Agree
- Undecided
- Disagree
- Strongly Disagree

1. **Telepharmacy can reduce patients' anxiety and stress.**

- Strongly Agree
- Agree
- Undecided
- Disagree
- Strongly Disagree

1. **Telepharmacy can facilitate the easy exchange of information with pharmacists and physicians.**

- Strongly Agree
- Agree
- Undecided
- Disagree
- Strongly Disagree

1. **Telepharmacy can make pharmaceutical services easier to pay for.**

- Strongly Agree
- Agree
- Undecided
- Disagree
- Strongly Disagree

1. **Telepharmacy can be better and easy scheduling revisits.**

- Strongly Agree
- Agree
- Undecided
- Disagree
- Strongly Disagree

1. **Telepharmacy can be easy communication and interaction with pharmacists.**

- Strongly Agree
- Agree
- Undecided
- Disagree
- Strongly Disagree

1. **Telepharmacy can be limited access to the internet connection / low bandwidth.**

- Strongly Agree
- Agree
- Undecided
- Disagree
- Strongly Disagree

1. **Technological literacy and skills are not required in Telepharmacy.**

- Strongly Agree
- Agree
- Undecided
- Disagree
- Strongly Disagree

***Section B: your preference for receiving pharmaceutical services***

1. **What is your preference for receiving pharmaceutical services?**

- Telepharmacy
- In-person visits to the pharmacy

1. **Why do you choose Telepharmacy or in-person visits to the pharmacy? (Please explain.)**
